# Impact of data source choice on multimorbidity measurement: a comparison study of 2.3 million individuals in the Welsh National Health Service

**DOI:** 10.1186/s12916-023-02970-z

**Published:** 2023-08-15

**Authors:** Clare MacRae, Daniel Morales, Stewart W. Mercer, Nazir Lone, Andrew Lawson, Emily Jefferson, David McAllister, Marjan van den Akker, Alan Marshall, Sohan Seth, Anna Rawlings, Jane Lyons, Ronan A. Lyons, Amy Mizen, Eleojo Abubakar, Chris Dibben, Bruce Guthrie

**Affiliations:** 1 Advanced Care Research Centre, University of Edinburgh, Bio Cube 1, Edinburgh BioQuarter, 13 Little France Road, Edinburgh, UK; 2 Usher Institute, College of Medicine and Veterinary Medicine, University of Edinburgh, Edinburgh, UK; 3 Division of Population Health and Genomics, University of Dundee, Dundee, UK; 4 Department of Public Health, University of Southern Denmark, Odense, Denmark; 5 Department of Public Health Sciences, Medical University of South Carolina, Charleston, USA; 6 Public Health, Institute of Health and Wellbeing, University of Glasgow, Glasgow, G12 9LX UK; 7 Institute of General Practice, Goethe University Frankfurt, Frankfurt Am Main, Germany; 8 Department of Public Health and Primary Care, Academic Center for General Practice, KU Leuven, Louvain, Belgium; 9 Department of Family Medicine, School CAPHRI, Maastricht University, Maastricht, The Netherlands; 10 School of Social and Political Science, University of Edinburgh, Chrystal Macmillan Building, Edinburgh, EH8 9LD UK; 11 School of Informatics, The University of Edinburgh, Edinburgh, UK; 12 Swansea University Medical School, Data Science Building, Singleton Campus, Swansea, UK; 13 University of Edinburgh Institute of Geography, Institute of Geography Edinburgh, Edinburgh, UK

**Keywords:** Electronic health records, Multimorbidity, Epidemiology

## Abstract

**Background:**

Measurement of multimorbidity in research is variable, including the choice of the data source used to ascertain conditions. We compared the estimated prevalence of multimorbidity and associations with mortality using different data sources.

**Methods:**

A cross-sectional study of SAIL Databank data including 2,340,027 individuals of all ages living in Wales on 01 January 2019. Comparison of prevalence of multimorbidity and constituent 47 conditions using data from primary care (PC), hospital inpatient (HI), and linked PC-HI data sources and examination of associations between condition count and 12-month mortality.

**Results:**

Using linked PC-HI compared with only HI data, multimorbidity was more prevalent (32.2% versus 16.5%), and the population of people identified as having multimorbidity was younger (mean age 62.5 versus 66.8 years) and included more women (54.2% versus 52.6%). Individuals with multimorbidity in both PC and HI data had stronger associations with mortality than those with multimorbidity only in HI data (adjusted odds ratio 8.34 [95% CI 8.02-8.68] versus 6.95 (95%CI 6.79-7.12] in people with ≥ 4 conditions). The prevalence of conditions identified using only PC versus only HI data was significantly higher for 37/47 and significantly lower for 10/47: the highest PC/HI ratio was for depression (14.2 [95% CI 14.1–14.4]) and the lowest for aneurysm (0.51 [95% CI 0.5–0.5]). Agreement in ascertainment of conditions between the two data sources varied considerably, being slight for five (kappa < 0.20), fair for 12 (kappa 0.21–0.40), moderate for 16 (kappa 0.41–0.60), and substantial for 12 (kappa 0.61–0.80) conditions, and by body system was lowest for mental and behavioural disorders. The percentage agreement, individuals with a condition identified in both PC and HI data, was lowest in anxiety (4.6%) and highest in coronary artery disease (62.9%).

**Conclusions:**

The use of single data sources may underestimate prevalence when measuring multimorbidity and many important conditions (especially mental and behavioural disorders). Caution should be used when interpreting findings of research examining individual and multiple long-term conditions using single data sources. Where available, researchers using electronic health data should link primary care and hospital inpatient data to generate more robust evidence to support evidence-based healthcare planning decisions for people with multimorbidity.

**Supplementary Information:**

The online version contains supplementary material available at 10.1186/s12916-023-02970-z.

## Background

Multimorbidity, most commonly defined as the coexistence of two or more long-term conditions, is an issue of global importance because of its association with increased healthcare use, mortality, and reduced quality of life [1]. Accurately estimating the prevalence of multimorbidity is therefore important to inform policy decision-making, healthcare planning, and research. The widespread availability of electronic health data, including electronic and administrative health records, presents opportunities for medical research examining multimorbidity, the volume of which has rapidly increased over the past two decades [2, 3].

Measurement of multimorbidity in research is highly variable in terms of the definition of multimorbidity used (for example two or more conditions, or three or more conditions from three or more body systems) [3, 4], the number and selection of conditions considered in the count [5], study setting, and participant age [6], resulting in widely varying estimates of the prevalence of multimorbidity [6]. Additionally, little is known about the impact of data source on multimorbidity research findings. A recent systematic review found that although many studies examining multimorbidity are based in primary care or community settings (441 of 566 [77.9%]), a lower proportion used electronic health records rather than patient self-report measures (142 of 441 [32.2%] in primary or community versus 89 of 103 [86.4%] in hospital settings respectively) [3].

Electronic health data are increasingly available for research, with 50% of upper-middle and high-income countries globally adopting these in primary and/or secondary care settings [7, 8]. Despite this, barriers to accessing electronic health data, particularly from primary care settings, can include access restrictions imposed by information governance legislation and challenges faced by researchers when manipulating and interpreting non-intuitive records of events and conditions [9]. Although the primary purpose of these data is to record the provision of clinical care, their large size and inclusion of populations often underrepresented in clinical trials and registries, such as women and people with multimorbidity [10], mean they better reflect true clinical populations [11]. However, routinely collected data may under-ascertain some conditions [12]. For example, using only primary care (PC) or only hospital inpatient (HI) data may lead to under-ascertainment of the prevalence and incidence of stroke [13] and myocardial infarction [12, 13]. It is unclear how general these findings are across the many conditions recommended for inclusion in studies of multimorbidity [14]. Despite the importance and widespread availability of these data, and the intensity of multimorbidity research, there is no standard approach to choice of data and little is currently understood about how the choice of data source impacts on the estimated prevalence of multimorbidity and its constituent conditions. The aim of this study was to compare the estimated prevalence of multimorbidity and the 47 constituent conditions using only PC, only HI, and linked PC-HI data in the SAIL Databank and to examine associations of condition counts derived in the same three ways with mortality.

## Methods

### Study design and population

This cross-sectional study used routinely collected anonymised data available in the SAIL Databank and consisted of individuals of all ages living in Wales and registered with a GP contributing data to the Secure Anonymised Information Linkage (SAIL) Databank on 1 January 2019. Intentionally, this study examines condition coding outside the COVID-19 pandemic to avoid capturing the effects of related restrictions and associated decreases in the diagnosis of physical and mental health conditions [15]. The study population was limited to people with at least 1 year of GP registration before 1 January 2019 to improve the stability of records and avoid under-ascertainment where an individual has recently moved practice and their PC record has not yet been populated with historic codes [16] and to those registered with GP practices who contribute data to SAIL Databank (80% of GP practices and 83% of Welsh residents [17]). The population was stratified into groups according to age, sex, and deprivation status of neighbourhood residence (using deciles of the Welsh Index of Multiple Deprivation [WIMD] 2019) [18]. Mortality was measured in the subsequent calendar year (to 31 December 2019).

### Data sources

PC data obtained from the Welsh Longitudinal General Practice Dataset (WLGP) were used to define conditions using Read version 2 codes (SNOMED-CT codes were not operational in the SAIL Databank during the study period), prescribing and/or laboratory data [19]. HI data were derived from general and psychiatric HI episodes obtained from the Patient Episode Database for Wales (PEDW) using all recorded International Classification of Diseases 10th Revision codes present for each hospital discharge [20]. PEDW records hospital inpatient events for English hospitals where a patient is registered with a Welsh GP; however, neither PEDW nor WLGP will provide data for patients prior to when they registered with a Welsh GP. Unlike Hospital Episode Statistics (HES) that records admissions, A&E attendances, and outpatient appointments from NHS England, PEDW records hospital inpatient episodes only [21]. Mortality data were derived from the Welsh Demographic Service Dataset.

### Definition of long-term conditions

Choice of the 47 conditions was based on results of a recent Delphi consensus study recommending those to include in the measurement of multimorbidity (Additional file 1) [14], and multimorbidity was defined as the presence of two or more conditions [1]. Phenotype definition and look-back duration for the codes defining each of the conditions followed rules defined by Barnett et al. [22] where possible. For the remaining conditions, inclusion criteria were agreed through discussion between authors CM, SWM, and BG. In certain cases, look-back durations varied within conditions to reflect the impact living with the condition was likely to have on an individual. For example, anaemia was defined as a relevant code ever recorded for aplastic anaemia, sickle cell anaemia, and thalassaemia (conditions that are either life-long or life-threatening), but as a relevant code dated in the last 12 months for iron-, B12-, or folate-deficient anaemias (conditions that are more likely to be transient), with the results of both combined into a single variable defining the presence of ‘anaemia’ on 1 January 2019. Unless the look-back duration was specifically stipulated, for example, 1 year for asthma clinical codes, codes present between 1 January 2000 and the study cross-section date of 1 January 2019 were used for both PC and HI data. This approach was taken to avoid relative over-ascertainment of PC codes. Historic codes are present for lifetime records that have been transcribed into the electronic record in the PC data source, but the first electronic records HI held within PEDW began on 1 April 1995. Code lists used to define conditions were those created by Kuan et al. [23] available on the HDR UK Phenotype Library [17] and de novo code lists created specifically by the authors of this study where required (detailed in Additional file 2). We adapted prescribing code lists from the Cambridge Multimorbidity Score by Payne et al. to qualify conditions that resolve as ‘active’ on 1 January 2019 (e.g. asthma, epilepsy) [24].

Prescribing and laboratory data were available within the PC datasource (WLGP). To ensure that the study reflected a fair comparison between ascertainment using codes present in PC and HI datasets based on availability within each data source, prescribing data were applied to only PC and to linked PC-HI data. Conditions were categorised by the International Classification of Diseases and Related Health Problems 10th Revision (ICD-10) (Additional file 2).

### Data analysis

We conducted a suite of analyses to estimate the prevalence and concordance of individual conditions and multimorbidity, and associations with mortality, between data sources. First, prevalence estimates for multimorbidity and each of the 47 conditions were calculated separately using only PC, only HI, and linked PC and HI (PC-HI) data. Second, the number of conditions each individual had was calculated using only PC, only HI, and linked PC-HI data. Associations with 12-month mortality were estimated using binary logistic regression and were used to calculate unadjusted and adjusted (by age, sex, and deprivation) odds ratios between morbidity counts (grouped into 0, 1, 2, 3, and 4 + conditions) with 95% confidence intervals. Third, PC/HI prevalence ratios were calculated by dividing the estimated prevalence measured using only PC data by the estimated prevalence measured using only HI data. Fourth, the proportion ascertained by each data source alone compared with linked PC-HI data was calculated, with Wilson’s exact method used to calculate 95% confidence intervals [25]. Finally, we estimated concordance between only PC and only HI data by [1] calculating the percentage of patients identified as having each of the 47 conditions in *both* PC and HI data (hereinafter referred to as ‘percent agreement’) and [2] calculating Cohen’s kappa for each individual condition and for multimorbidity, using the following formula [26]:

$$Kappa = \left({p}_{o}-{p}_{h}\right) /\left(1-{p}_{h}\right)$$

where:

*p*_o_: Relative observed agreement among PC and HI data

*p*_h_: Hypothetical probability of chance agreement between PC and HI data.

Kappa statistic for each of the 47 conditions was stratified into categories to describe concordance between data sources (slight 0.01–0.2, fair 0.21–0.40, moderate 0.41–0.60, substantial 0.61–0.8, almost perfect 0.81–1.00). Given that HI ascertainment of asthma and epilepsy was not constrained by prescribing data but PC and linked PC-HI was, the final three measures of concordance could not be assessed for these conditions.

The project received ethical approval from the SAIL Databank independent information governance panel [27]. Data cleaning was performed using SQL to query IBM DB2 databases. Analysis, performed using the glm function in ‘stats’ package, and data visualisation were performed using R version 4.1.2 [28].

### Role of funding source

The funders of the study had no role in the study design, data collection, data analysis, data interpretation, or writing of the report. The corresponding author had full access to all the data used in the study and had final responsibility for the decision to submit the study for publication.

## Results

On 1 January 2019, 2,340,027 individuals living in Wales were registered with SAIL-contributing GP practices for at least 1 year. Multimorbidity had the highest estimated prevalence using linked PC-HI data (32.2%), followed by only PC data (29.6%), and lowest using only HI data (16.5%) (Table 1). The mean age of people with multimorbidity was nearly 4 years younger using linked PC-HI data (62.5 years) compared with only HI data (66.8 years). The proportion of women with multimorbidity was nearly two percentage points higher using linked PC-HI data (54.2% [408,760 of 754,082]) than HI data (52.7% [202,678 of 385,276]). There was little difference in the distribution of people by deprivation status when multimorbidity was defined using different data sources (Table 1).

**Table 1:** Study population characteristics for the whole study cohort and by multimorbidity measured using different data sources

	**All cohort**	**No. (%) of people in row with multimorbidity**		
		**Only primary care (PC) data**	**Only hospital inpatient (HI) data**	**Linked primary care/hospital inpatient data**
Whole cohort	2,340,027	691,868 (29.6)	385,276 (16.5)	754,082 (32.2)
Age, years				
Mean (SD)		63.0 (17.7)	66.8 (16.7)	62.5 (17.5)
0–4	83,487	201 (0.2)	191 (0.2)	350 (0.4)
5–9	137,384	1113 (0.8)	740 (0.5)	1593 (1.2)
10–14	137,510	2235 (1.6)	1157 (0.8)	2808 (2.0)
15–19	125,153	4864 (3.9)	2006 (1.6)	5802 (4.6)
20–29	272,938	25,999 (9.5)	9954 (3.6)	29,902 (10.9)
30–39	292,464	41,954 (14.3)	16,176 (5.5)	47,662 (16.3)
40–49	293,387	66,470 (22.7)	26,054 (8.9)	74,168 (25.3)
50–59	339,737	116,750 (34.4)	52,378 (15.4)	128,917 (37.9)
60–69	285,149	149,251 (52.3)	80,838 (28.3)	162,044 (56.8)
70–79	235,988	166,920 (70.7)	107,090 (45.4)	178,513 (75.6)
80–89	112,276	94,674 (84.3)	71,415 (63.6)	99,854 (88.9)
90 +	24,554	21,437 (87.3)	17,277 (70.4)	22,469 (91.5)
Sex				
Men and boys	1,167,242	315,460 (27.0)	182,598 (15.6)	345,322 (29.6)
Women and girls	1,172,785	376,408 (32.1)	202,678 (17.3)	408,760 (34.8)
Deprivation				
1 (most)	252,310	75,612 (30.0)	43,263 (17.1)	81,862 (32.4)
2	242,936	73,863 (30.4)	42,056 (17.3)	80,307 (33.0)
3	231,456	72,979 (31.5)	41,172 (17.8)	78,783 (34.0)
4	248,863	73,613 (29.6)	41,510 (16.7)	80,495 (32.3)
5	233,634	67,087 (28.7)	37,949 (16.2)	73,637 (31.5)
6	220,335	65,455 (29.7)	36,025 (16.3)	71,309 (32.4)
7	223,109	64,937 (29.1)	36,382 (16.3)	71,419 (32.0)
8	215,813	64,790 (30.0)	34,868 (16.1)	70,210 (32.5)
9	232,343	66,741 (28.7)	36,195 (15.6)	72,910 (31.4)
10 (least)	239,228	66,791 (27.9)	35,856 (15.0)	73,150 (30.6)

**Fig. 1:**
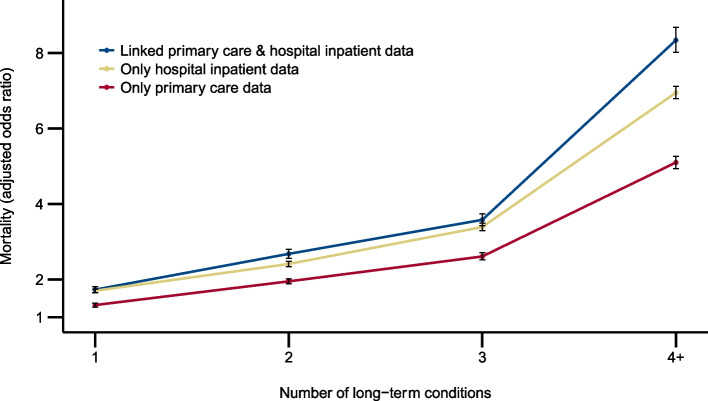
Adjusted odds ratios for 12-month mortality. Odds ratios for 12-month mortality in people with 4 + versus 0 conditions, adjusted for age, sex, and deprivation status. Ninety-five percent confidence intervals are represented by error bars

The 1-year mortality rate increased markedly with increasing number of conditions when identified in all three data sources (Additional file 3). In unadjusted analysis, the odds ratio for mortality in people with 4 + versus those with 0 conditions was 43.83 (95%CI 42.63 to 45.07) where conditions were identified only in PC, 43.10 (95%CI 42.20 to 44.03) where conditions were identified only in HI, and 69.35 (95%CI 66.92 to 71.87) where conditions were identified in both PC and HI data (Additional file 3). Adjusting for age, sex, and deprivation, the odds ratio for mortality in people with 4 + versus 0 conditions was 5.10 (95%CI 4.94 to 5.26) for PC, 6.95 (95%CI 6.79 to 7.12) for HI, and 8.34 (95%CI 8.02 to 8.68) for both PC and HI data (Fig. 1 and Additional file 3).

**Fig. 2:**
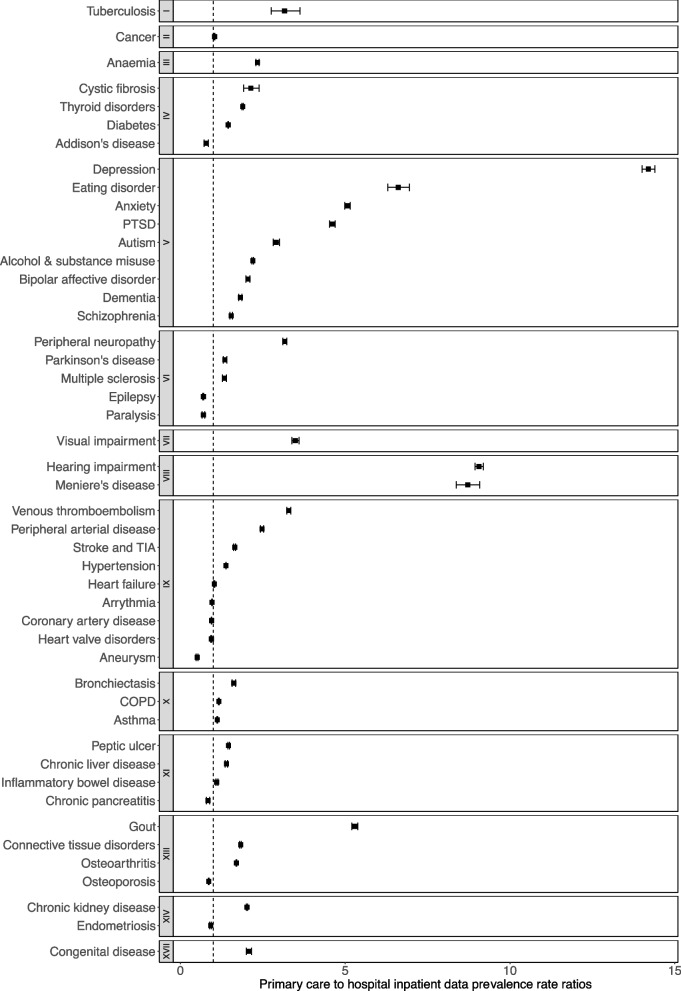
Forest plot of primary care to hospital inpatient data prevalence ratios. Prevalence ratios are calculated by dividing prevalence using only primary care (PC) data by prevalence using only hospital inpatient (HI) data: PC/HI ratio. Error bars represent 95% confidence intervals. The vertical dotted line represents where the PC/HI ratio is 1, meaning the prevalence rate is the same using both PC and HI data. Where the ratio is > 1, the prevalence was higher using PC versus HI data. Conversely, a ratio < 1 represents conditions where prevalence is higher using HI versus PC data. Conditions are grouped by ICD-10 chapter

The estimated prevalence of most conditions was higher using only PC versus only HI data (PC/HI prevalence ratios). For 37/47 conditions, the PC/HI data prevalence ratio was statistically significantly > 1 (i.e. prevalence using only PC > only HI) including tuberculosis, cancer, and anaemia; congenital disease, visual impairment, and all mental and behavioural disorders; diseases of the respiratory system; and diseases of the ear and mastoid process. The PC/HI prevalence ratios were statistically significantly lower for 10/47 conditions: Addison’s disease, epilepsy, paralysis, coronary artery disease, heart valve disorders, arrythmia, aneurysm, osteoporosis, and endometriosis (Fig. 2 and Additional file 5). Conditions with the highest PC/HI prevalence ratios were mental and behavioural and sensory disorders (depression at 14.2 [95% CI 14.0 to 14.4] and hearing impairment at 9.1 [95% CI 8.9, 9.2]) and lowest were diseases of the circulatory system and the nervous system (aneurysm at 0.5 [95% CI 0.5 to 0.5] and paralysis at 0.7 [95% CI 0.7 to 0.7]). The PC/HI prevalence ratios were close to 1 (lying between 0.9 and 1.1) for six conditions (cancer, arrythmias, coronary heart disease, heart failure, heart valve disorders, and endometriosis) of which four were diseases of the circulatory system (Table 2).

**Table 2:** Prevalence of long-term conditions using primary care, hospital inpatient, and linked primary care to hospital inpatient data

ICD-10 chapter	Long-term condition	Prevalence, no. (%)			Difference between PC and HI, number (% of total cohort)
		**Primary care data**	**Hospital inpatient data**	**Linked PC-HI data**	
I—Certain infectious and parasitic diseases	Tuberculosis	876 (0.04)	277 (0.01)	1030 (0.40)	599 (0.03)
II—Neoplasms	Cancer	41,905 (1.79)	40,284 (1.72)	53,315 (2.28)	1621 (0.07)
III—Diseases of blood/blood-forming organs	Anaemia	30,040 (1.28)	12,833 (0.55)	39,535 (1.69)	17,207 (0.74)
IV—Endocrine, nutritional, and metabolic diseases	Cystic fibrosis	1002 (0.04)	468 (0.02)	966 (0.04)	534 (0.02)
	Thyroid disorders	121,217 (5.18)	64,041 (2.74)	127,776 (5.46)	57,176 (2.44)
	Addison’s disease	1075 (0.05)	1371 (0.06)	1689 (0.07)	− 296 (− 0.01)
	Diabetes	149,527 (6.39)	103,211 (4.41)	159,495 (6.82)	46,316 (1.98)
V—Mental and behavioural disorders	Depression	305,915 (13.07)*	21,540 (0.92)	311,518 (13.31)*	284,375 (12.15)
	Eating disorder	11,830 (0.51)	1789 (0.08)	12,399 (0.53)	10,041 (0.43)
	Anxiety	92,622 (3.96)*	18,254 (0.78)	106,024 (4.53)*	74,368 (3.18)
	PTSD	12,008 (0.51)	2600 (0.11)	13,340 (0.57)	9408 (0.40)
	Autism	14,283 (0.61)	4907 (0.21)	15,635 (0.67)	9376 (0.40)
	Alcohol and substance misuse	81,830 (3.50)	37,252 (1.59)	96,610 (4.13)	44,578 (1.91)
	Bipolar affective disorder	13,437 (0.57)*	6548 (0.28)	15,533 (0.66)*	6889 (0.29)
	Dementia	15,817 (0.68)	8689 (0.37)	18,171 (0.78)	7128 (0.30)
	Schizophrenia	14,124 (0.60)	9195 (0.39)	16,792 (0.72)	4929 (0.21)
VI—Diseases of the nervous system	Peripheral neuropathy	48,462 (2.07)	15,280 (0.65)	57,226 (2.45)	33,182 (1.42)
	Parkinson’s disease	5047 (0.22)	3735 (0.26)	5983 (0.26)	1312 (0.06)
	Multiple sclerosis	4718 (0.20)	3520 (0.15)	5136 (0.22)	1198 (0.05)
	Epilepsy	20,789 (0.89)*	25,577 (1.09)	22,348 (0.96)*	− 7684 (− 0.33)
	Paralysis	1811 (0.08)	2586 (0.11)	3382 (0.14)	− 775 (− 0.03)
VII—Diseases of the eye and adnexa	Visual impairment	18,450 (0.79)	5288 (0.23)	20,990 (0.90)	13,162 (0.56)
VIII—Diseases of the ear and mastoid process	Hearing impairment	207,245 (8.86)	22,865 (0.98)	216,682 (9.26)	184,380 (7.88)
	Meniere’s disease	22,661 (0.97)	2600 (0.11)	23,461 (1.00)	20,061 (0.86)
IX—Diseases of the circulatory system	Venous thromboembolism	51,509 (2.20)	15,657 (0.67)	54,907 (2.35)	35,852 (1.53)
	Peripheral artery disease	30,863 (1.32)	12,446 (0.53)	35,303 (1.51)	18,417 (0.79)
	Stroke and TIA	62,292 (2.66)	37,853 (1.62)	69,889 (2.99)	24,439 (1.04)
	Hypertension	402,322 (17.19)	290,995 (12.44)	456,687 (19.52)	111,327 (4.76)
	Heart failure	31,295 (1.34)	30,499 (1.30)	45,508 (1.94)	796 (0.03)
	Coronary artery disease	101,163 (4.32)	106,741 (4.56)	127,650 (5.46)	− 5578 (− 0.24)
	Heart valve disorders	29,263 (1.25)	31,152 (1.33)	45,931 (1.96)	− 1889 (− 0.08)
	Arrythmia	68,841 (2.94)	71,366 (3.05)	88,746 (3.79)	− 2525 (− 0.11)
	Aneurysm	2550 (0.11)	5048 (0.22)	6143 (0.26)	− 2498 (− 0.11)
X—Diseases of the respiratory system	Bronchiectasis	9040 (0.39)	5567 (0.24)	10,930 (0.47)	3473 (0.15)
	COPD	64,083 (2.74)	54,815 (2.34)	84,075 (3.59)	9268 (0.40)
	Asthma	203,049 (8.68)*	181,470 (7.76)	210,633 (9.00)*	21,579 (0.92)
XIII—Diseases of the musculoskeletal system	Gout	83,002 (3.55)	15,692 (0.67)	85,330 (3.65)	67,310 (2.88)
	Connective tissue disorders	56,345 (2.41)	30,870 (1.32)	64,571 (2.76)	25,475 (1.09)
	Osteoarthritis	243,414 (10.40)	142,901 (6.11)	293,678 (12.55)	100,513 (4.30)
	Osteoporosis	55,044 (2.35)	63,705 (2.72)	98,677 (4.22)	− 8661 (− 0.37)
XI—Diseases of the digestive system	Peptic ulcer	33,788 (1.44)	23,095 (0.99)	45,293 (1.94)	10,693 (0.46)
	Chronic liver disease	13,804 (0.59)	9881 (0.42)	17,903 (0.77)	3923 (0.17)
	Inflammatory bowel disease	20,016 (0.86)	18,187 (0.78)	24,037 (1.03)	1829 (0.08)
	Chronic pancreatitis	2038 (0.09)	2425 (0.10)	3405 (0.15)	− 387 (− 0.02)
XIV—Diseases of the genitourinary system	Chronic kidney disease	110,493 (4.72)	54,677 (2.34)	131,348 (5.61)	55,816 (2.39)
	Endometriosis	12,210 (0.52)	13,305 (0.57)	18,080 (0.77)	− 1095 (− 0.05)
XVII—Congenital malformations	Congenital disease	7282 (0.31)	3502 (0.15)	8176 (0.35)	3780 (0.16)

^*^Primary care (PC) and linked PC_HI prevalence estimates include prescribing data as documented in Additional File 2

For most conditions, using only PC data ascertained a higher proportion of people identified using linked PC-HI data than using only HI data. The largest disparity between only PC/linked PC-HI versus only HI/linked PC-HI was seen for diseases of the ear and mastoid process (median 96.1% [IQR 95.9 to 96.3] versus 10.8% [95% CI 10.7 to 10.9]) and mental and behavioural disorders (median 87.4% [IQR 86.5 to 95.4] versus 31.4% [IQR 17.2 to 42.2]) respectively. In contrast, conditions in diseases of the circulatory system (79.2% [IQR 68.8 to 88.1] versus 67.0% [IQR 54.2 to 80.4]) and neoplasms (78.6% [95% CI 78.2 to 78.9] versus 75.6% [95% CI 75.2 to 75.9]) had the smallest difference. Compared with linked HI-PC data, only PC data ascertained a larger proportion than only HI on averaging across all conditions: median ascertainment using only PC data was 84.7% (IQR 76.7 to 90.4) versus 50.1% (IQR 31.4 to 67.0) for only HI data (Additional file 4 and Additional file 5).

**Fig. 3:**
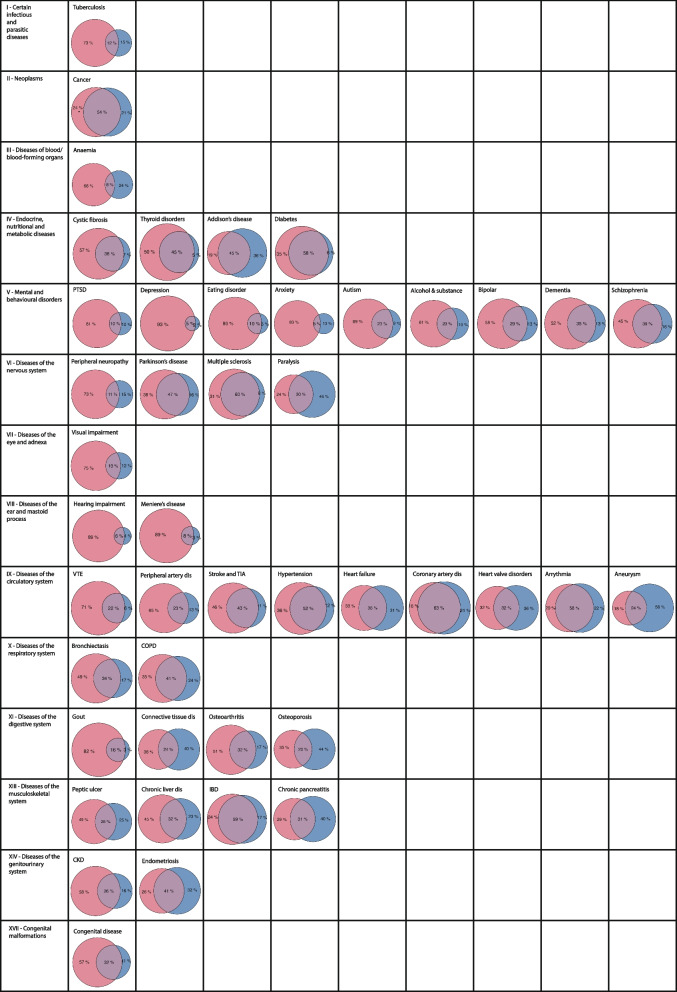
Venn diagrams of concordance between data sources for 47 long-term conditions by ICD-10 body system. Red represents people identified as having each condition in primary care (PC) and blue represents people identified in hospital inpatient (HI) datasources. Area of cross-over represents individuals identified as having each condition in both data sources

Concordance between data sources was variable across conditions and ICD-10 body systems. The percentage agreement of people identified as having each condition in both PC and HI data varied considerably across conditions, ranging from a minimal agreement in anxiety (4.6%) and depression (5.1%) to a maximal agreement in coronary artery disease and (62.9%) and multiple sclerosis (60.4%). ICD-10 chapters with the highest percent agreement were endocrine, nutritional, and metabolic diseases (median 44.9% [IQR 42.6 to 48.4]) and diseases of the nervous system (median across conditions in that chapter 38.10% [IQR 25.3 to 49.7]). Percent agreement was lowest for diseases of the ear and mastoid process (median 6.9% [IQR 6.6 to 7.3]) and mental and behavioural disorders (median 22.7% [IQR 5.1 to 28.7]) (Fig. 3 and Additional file 5). Agreement measured using Cohen’s kappa was slight (< 0.20) for five, fair (0.21–0.40) for 12, moderate (0.41–0.60) for 16, and substantial (0.61–0.80) for 12 conditions. Kappa was lowest in depression (0.08) and hearing impairment (0.10), and highest in diabetes (0.72) and alcohol and substance misuse (0.79). At the ICD-10-chapter level, conditions with the lowest kappa were found in mental and behavioural disorders (three slight and two fair agreement out of nine) and diseases of the ear and mastoid process (two slight out of two); in contrast, ICD-10 chapters with the highest kappa were seen in diseases of the circulatory system (four substantial and two moderate out of nine) and diseases of the endocrine system (three substantial and one moderate out of four)

## Discussion

The prevalence of multimorbidity was higher using only PC (29.6%) than only HI (16.5%) data and higher still using linked PC-HI (32.2%) data. The population of people identified as having multimorbidity using linked PC-HI data compared to only HI was younger, included a higher proportion of women, and people identified as multimorbid in both PC and HI had a stronger association with mortality. Using only PC data identified more people as having most of the 47 conditions than using only HI, and this was most marked for mental and behavioural and sensory disorders. Concordance between data sources was variable across conditions and ICD-10 body systems. The use of single data sources may underestimate the prevalence of multimorbidity and most individual conditions, especially mental and behavioural disorders. Findings from this study support the use of linked primary care and hospital inpatient data where available.

Strengths of the study are the inclusion of almost the entire adult population of Wales and examination of multimorbidity and a large number of individual conditions recommended for use in multimorbidity research [14], providing granular insights into variation in the relative ascertainment of disease from PC versus HI data sources. Limitations include variation in longitudinal availability of data for individuals (for example because individuals change GP registration or migrate into Wales), although we mitigated against this by requiring 1 year of GP registration to minimise impact [16]. Like other UK datasets, PEDW data only reliably includes ICD-10 codes for hospital inpatient events, although all specialist outpatient clinics generate a letter to the general practitioner which is commonly used to code the primary care data. A further limitation is that primary care prescribing data were used to qualify epilepsy and asthma as ‘active’ on the analysis date, with the same data/rules used to estimate prevalence in linked PC-HI data. In contrast, only HI data were not qualified in this way, meaning that ascertainment of asthma and epilepsy are not strictly comparable because only PC and linked PC-HI estimates are for ‘active’ disease, whereas only HI is for ‘ever recorded’. Finally, we treated the linked PC-HI estimates of prevalence as gold standard but did not have any way of examining false positive diagnoses in either of the data sources.

Consistent with our findings, a study from Canada found varying degrees of discordance comparing ascertainment of seven conditions (myocardial infarction, asthma, diabetes, chronic lung disease, stroke, hypertension, and congestive heart failure) between the Canadian Community Health Survey (patient self-report) and health administrative data [29]. Ascertainment of diabetes and hypertension were similar, but administrative health data gave lower prevalence estimates for stroke, congestive heart failure, and COPD. Another study, from the USA, compared ascertainment of conditions using hospital outpatient EHRs and encounter diagnosis data from Community Health Center (CHC) patients, where care is provided for un- and under-insured patients regardless of their ability to pay, found considerable variation in ascertainment across sources [30]. They conclude that using EHRs capturing hospital outpatient data only might under-ascertain conditions in people who attend CHCs with less access to hospital services. In our study, where we have examined a broader range of conditions in individuals who have access to universal care that is free at the point of delivery, using hospital inpatient data alone usually under-ascertains conditions, most consistently for mental health conditions. Given marked socioeconomic gradients in mental-physical health multimorbidity, it is important to ascertain mental and behavioural disorders to represent morbidities experienced by people living in deprived areas [4], who often have poorer health outcomes [31]. This is important in terms of application to health policy where models predicting healthcare costs and risk of admission perform better when two sources, outpatient and prescribing data, are linked than when using single sources [32].

It is important to note the variation in prevalence estimates for individual conditions and multimorbidity where differing rules for ascertainment have been applied across studies. Ascertainment of conditions using the same criteria can be similar; for example, estimates of hypertension prevalence calculated as any time look-back for clinical codes were 19.52% in this study compared with 18.2% in a recent study using linked primary care to HES in CPRD (both studies examined all ages) [5]. However, where ascertainment rules differ, such as when ascertaining depression, estimated prevalence was lower (13.31%) in the current study where a 1-year look-back for either clinical codes or prescribing activity was necessary to reach the diagnosis compared with a higher estimate of 17.3% in the recent study where any time look-back for codes was used [5]. Multimorbidity prevalence estimates and associations with adverse outcomes vary across studies where studies use different data sources and numbers and selections of conditions. For example, in the current study, the aOR for mortality was 8.34 (95% CI 8.02,8.68) when using linked PC-HI data. This is higher than a recent similar study also in SAIL Databank using primary care data to define the 40 long-term conditions described by Barnett et al. [22] who report a hazard ratio of 5.14 (95% CI 4.95–5.34) for mortality in people with five or more long-term conditions [33] and although the aOR in the current study cannot be directly compared with a HR the result was similar when using PC only data (5.10 [95%CI 4.94-5.26]).

Similar to the 14-fold higher ascertainment of depression using only PC versus only HI data in this study, previous studies have shown that the recording of depression in hospital data is incomplete and has been attributed to clinicians considering depression as being non-relevant to admissions for physical conditions [34]. A similar pattern is seen in studies examining ascertainment of musculoskeletal conditions in hospital data, in particular where clinicians under-reported back pain [35]. In this study, osteoarthritis was 1.7 times more commonly identified from only PC than only HI data, although both may under-ascertain because patients can under-consult with this condition to medical professionals because they consider it to be part of the normal ageing process [36]. There was substantial agreement between data sources for several conditions, including inflammatory bowel disease where this is likely to reflect the need for shared primary and hospital care, and the frequency of hospital admission for acute disease flares [37].

The implication of the study for clinicians and managers is that coding of conditions in the two settings seems inconsistent, reflecting often a manual transfer of diagnoses between settings. More consistent coding is important to improve information transfer across the primary care-hospital boundary, which is a critical underpinning for good care. It will be necessary to examine the effects of the implementation of SNOMED-CT codes in electronic health records in the UK. Due to the use of a more consistent medical vocabulary, it is anticipated that the introduction of SNOMED-CT will improve precision in the exchange of clinical information between primary and secondary care settings for both clinical and research purposes and therefore comparability with international studies where the same system is used [38]. For researchers, the key implication is to recognise that only using hospital data is likely to seriously under-ascertain many conditions (although it will identify people with more severe disease for some conditions like heart failure), which will particularly matter in studies of multimorbidity or in studies where mental health is important. There are, however, certain circumstances where it is appropriate to use primary care data alone, for example when examining trajectories of workload pressures in primary care [39] or changes in quality of care in relation to financial incentives offered to general practitioners [40]. Further research is needed to examine the validity of diagnoses recorded in both primary and secondary care, with accurate estimation of false positive and false negative rates for different choices of data source. For multimorbidity studies involving large numbers of conditions, it is unlikely that gold-standard medical data review at the scale requires is feasible, but code lists can be at least partially validated by examining associations with a range of other data including clinical outcomes, laboratory, and prescribing data.

## Conclusions

This study highlights the importance of, where available, linking primary care and hospital inpatient data when measuring multimorbidity to avoid underestimation of prevalence and underrepresentation of certain population groups. Robust and consistent methods across studies are needed to improve comparability and reproducibility and ultimately improve the quality of research and the clinical trials and guideline development needed to support people with multimorbidity.

### Supplementary Information

**Additional file 1:** Choice of long-term conditions in relation to those included by “Measuring multimorbidity in research: a Delphi consensus study” (Ho et al. 2022).

**Additional file 2:** List of included long-term conditions including explanation of rules and code lists used to define Conditions.

**Additional file 3:** Associations between long-term conditions and mortality by data source.

**Additional file 4:** Measures of concordance of long-term conditions using primary care and hospital inpatient data.

**Additional file 5:** Measures of concordance of long-term conditions using different data sources.

## Data Availability

The datasets that will be used are available in an anonymised form via a secure data-sharing platform, underpinned by the ISO 27001 internationally recognised best practice standard for an Information Security Management System, and compliant with National Research Ethics Service guidance. Access to the data is available only for accredited researchers using a secure remote desktop login and following approval for a project via an application to the SAIL IGRP (https://saildatabank.com/governance/). All data released from the SAIL Databank safe haven is available within the Additional files. Potentially disclosive data can be accessed by applying to the original data holders reported in the ‘ Methods’ section.

## Abbreviations

A&E: Accident and emergency
HDR UK: Health Data Research UK
HES: Hospital Episode Statistics
HI: Hospital inpatient
ICD-10: International Classification of Diseases and Related Health Problems 10th Revision
Linked PC-HI: Linked primary care to hospital inpatient data
NHS: National Health Service
PC: Primary care
PEDW: Patient Episode Database for Wales
SAIL: Secure Anonymised Information Linkage
SNOMED-CT: Systematized Nomenclature of Medicine Clinical Terms
WIMD: Welsh Index of Multiple Deprivation
WLGP: Welsh Longitudinal General Practice Dataset
