# Supplementary material for: Impact of data source choice on multimorbidity measurement: a comparison study of 2.3 million individuals in the Welsh National Health Service
Source: BMC Med. 2023 Aug 15;21:309. doi: 10.1186/s12916-023-02970-z (PMC10426056; doi:10.1186/s12916-023-02970-z)
Supplement: Supplementary file 2 — Additional file 2: List of included long-term conditions including explanation of rules and code lists used to define Conditions. [file 12916_2023_2970_MOESM2_ESM.docx]

# Additional File 2. List of included long-term conditions including explanation of rules and code-lists used to define conditions.

| **Condition name (Delphi derivatives where conditions are lumped)** | **Barnett et al^1^ rules** | **Response to Barnett rules** | **PC implementation rules** | **HI implementation rules** | **Linked PC-HI implementation rules** | **Code list or code list Read v2***^,^** | **Code list or code list ICD-10***^,^** |
| --- | --- | --- | --- | --- | --- | --- | --- |
| Addison’s disease | Not included | NA | Read v2 code ever recorded | ICD-10 code ever recorded | Read v2 or ICD-10 code ever recorded | A176., C154., C1540, C1541, C1540, C154z | E271, E272, E271, E274 |
| Alcohol and substance misuse | Read v2 code ever coded | Unchanged | Read v2 code ever recorded | ICD-10 code ever recorded | Read v2 or ICD-10 code ever recorded | c945 - alcohol misuse,  c1594 - substance misuse | c947 - alcohol misuse,  1596 - substance misuse |
| Anaemia | Not included | NA | Read v2 code in the previous 12-months (iron deficiency, B12 deficiency, folate deficiency), Read v2 code ever recorded (aplastic anaemia, sickle cell anaemia, thalassaemia) | ICD-10 code in the previous 12-months (iron deficiency, B12 deficiency, folate deficiency), ICD10 code ever recorded (aplastic anaemia, sickle cell anaemia, thalassaemia) | Read v2 or ICD10 code 12-month look back (iron deficiency, B12 deficiency, folate deficiency), Read v 2 or ICD10 code ever recorded (aplastic anaemia, sickle cell anaemia, thalassaemia) | \| c831 - iron deficiency anaemia, c979 - aplastic anaemia, c1013 - B12 deficiency anaemia, c1174 - folate deficiency anaemia, c1556 - sickle cell anaemia, c1603 - thalassaemia \| \| --- \| | c833 - iron deficiency anaemia, c981 - aplastic anaemia, c1015 - B12 deficiency anaemia, c1176 - folate deficiency anaemia, c1558 - sickle cell anaemia, c1602 - thalassaemia |
| Aneurysm | Not included | NA | Read 2 code ever recorded | ICD-10 code ever recorded | Read v2 or ICD-10 code ever recorded | c783 - abdominal aortic aneurysm | c785 - abdominal aortic aneurysm |
| Anxiety | Read code in last 12 months | Unchanged | Read v2 code in the previous 12-months OR 4 or more anxiolytic/hypnotic prescriptions in the previous 12-months | Read v2 code in the previous 12-months | Read v2 or ICD-10 code in the previous 12-months OR 4 or more anxiolytic/hypnotic prescriptions in the previous 12-months | c976 - anxiety | c978 - anxiety |
| Arrythmia | Read code ever coded (atrial fibrillation only) | Unchanged | ICD-10 code ever recorded | Read v2 code ever recorded | Read v2 or ICD-10 code ever recorded | c789 - atrial fibrillation, c915 – supraventricular tachycardia, c1010 – atrioventricular block complete  c1553 - sick sinus syndrome, c1615 - trifasicular block | c791 - atrial fibrillation, c917 – supraventricular tachycardia, c1012 – atrioventricular block complete, c1555 - sick sinus syndrome, c1617 - trifasicular block |
| Asthma | Read code ever coded | Unchanged | Read v2 code ever recorded AND any prescription in the previous 12-months | ICD-10 code ever recorded | Read v2 or ICD-10 code ever recorded AND any prescription in the last 12 months AND no Read v2 or ICD-10 Chronic Obstructive Pulmonary Disease code ever recorded | c2418 - asthma | c994 - asthma |
| Autism | Not included | NA | Read 2 code ever recorded | ICD-10 code ever recorded | Read v2 or ICD-10 code ever recorded | c995 - autism | c997 - autism |
| Bipolar affective disorder | Read v2 code ever coded | Unchanged | Read v2 code ever recorded OR lithium ever prescribed | ICD-10 code ever recorded | Read v 2 or ICD-10 code ever coded OR lithium ever prescribed | c793 - bipolar | c795 - bipolar |
| Bronchiectasis | Read v2 code ever coded | Unchanged | Read 2 code ever recorded | ICD-10 code ever recorded | Read v2 or ICD-10 code ever recorded | c1045 - bronchiectasis | c1047 - bronchiectasis |
| Coronary artery disease | Read v2 code ever coded | Unchanged | Any ICD-10 code ever recorded | Any Read v2 code ever recorded | Any Read v2 or ICD-10 code ever recorded | c802 – coronary heart disease,  c1296 – myocardial infarction,  c1588 - stable angina,  c1631 - unstable angina | \| c804 – coronary heart disease,  c1298 – myocardial infarction,  c1590 - stable  angina,  c1633 – unstable  angina \| c1590 - stable angina \| c1633 - unstable angina \| \| --- \| --- \| --- \| |
| Cancer (Delphi: Solid organ cancers, haematological cancer, metastatic cancer, melanoma) | Read code (first) coded in the last 5 years | Unchanged | First Read v2 code in the previous 12-months | First ICD-10 code in the previous 12-months | First Read v2 or ICD-10 code in the previous 12-months | c846 – myelodysplastic, c858 - non-Hodgkin’s lymphoma, c1218 - Hodgkin’s lymphoma, c1258 – leukaemia, c1361 - primary myeloma, c1415 - primary melanoma, c1385 - primary biliary, c1388 - primary bladder, c1391 - primary bone, c1397 - primary brain, c1400 - primary breast, c1403 - primary cervical, c1406 - primary kidney, c1409 - primary liver, c1412 - primary lung, c1418 - primary mesothelioma, c1421 - primary multiple, c1424 - primary oesophageal, c1430 - primary other, c1433 - primary ovarian, c1436 - primary pancreatic, c1439 - primary prostate, c1445 - primary stomach, c1448 - primary testicular, c1454 - primary thyroid, c1457 - primary uterine, c1513 - secondary lymph nodes, c1516 - secondary adrenal, c1519 - secondary bone, c1522 - secondary bowel, c2065 - bowel cancer primary or secondary, c1525 - secondary brain, c1528 - secondary liver, c1531 - secondary lung, c1540 - secondary peritoneum, c1543 - secondary pleura, c1537 - secondary other | c848 – myelodysplastic, c860 - non-Hodgkin’s lymphoma, c1220 - Hodgkin’s lymphoma, c1260 – leukaemia, c1363 - primary myeloma, c1417 - primary melanoma, c1387 - primary biliary, c1390 - primary bladder, c1393 - primary bone, c1399 - primary brain, c1402 - primary breast, c1405 - primary cervical, c1408 - primary kidney, c1411 - primary liver, c1414 - primary lung, c1420 - primary mesothelioma, c1423 - primary multiple, c1426 - primary oesophageal, c1432 - primary other, c1435 - primary ovarian, c1438 - primary pancreatic, c1441 - primary prostate, c1447 - primary stomach, c1450 - primary testicular, c1450 - primary thyroid, c1459 - primary uterine, c1515 - secondary lymph nodes, c1518 - secondary adrenal, c1521 - secondary bone, c1524 - secondary bowel, c1527 - secondary brain, c1530 - secondary liver, c1533 - secondary lung, c1542 - secondary peritoneum, c1545 - secondary pleura, c1539 – secondary other |
| Cystic fibrosis | Not included | NA | Any Read v2 code ever recorded  AND NO bronchiectasis Read v2 code every recorded | Any ICD-10 code ever recorded  AND NO bronchiectasis ICD-10 code every recorded | Any Read v2 or ICD-10 or Read v2 code ever recorded  AND NO bronchiectasis Read v2 or ICD-10 code ever recorded | C799 – cystic fibrosis | c801 - cystic fibrosis |
| Chromosomal abnormalities† | Not included | NA | Any Read v2 code ever recorded | Any ICD-10 code ever recorded | Any Read v2 or ICD-10 code ever recorded | PJ00., PJ01., PJ01., PJ0.., PJ0.., PJ0z., PJ0z., PJ0.., PJ02., PJ02., PJ20., PJ21., PJ21., PJ2.., PJ2z., PJ2z., PJ22., PJ10., PJ11., PJ11., PJ1.., PKyz0, PJ1z., PJ1z.,  PJ12., PJ50w, PJ50x, PJ50x, PJ510, PJ511, PJ520, PJ521, PJ523, PJ524, PJ513, PJ513, PJ515, PJ515, PJ512, PJ503, PJ514, PJ514, PJ500, PJ501, PJ502, PJ503, PJ504, PJ505, PJ506, PJ0.., PJ508, PJ50., PJ51., PJ51z, PJz3., PJ50z, PyuA0, PJ507, PJ50y, PJ52., PJ52z, PJ36., PJ370, PJ37., PJ37z, PJ37., PJ38., PJ32., PJ31., PJ338, PJ339, PKyz7, PKyz5, PKyz7, PKyz5, PJ330, PJ30., PJz2., PJ3z., PJ30.11, PyuA1, PJ33., PJ33z, PJ3y., PJ331, PJ332, PJ331, PJ332, PJ332, PJ331, PJ333, PJ336, PJ337, PJ334, PJ34., PJ35., PJ3.., PyuA2, PJ37., PJ531, PJ632, PJ633. PJ60., PJy10, PJ635, PJ630, PJ636, PJ634, PJ63., PJ63z, PJ63z, PyuA5, PJ636, PJ63z, PJ6.., PJ6z., PJ64., PJ64z, PJ62., PJ631, PJy2.  PJy2., PJy4., PJy5..PJ640, PyuA6, PJy12, PJyy0, PJyy1, PJyy4, PJyy2, F1y0., PyuAB, PJy1z, PJy1., PJy13, PJyy., PyuAD | Q968, Q969, Q97, Q970, Q971, Q972, Q973, Q978, Q979, Q980, Q99, Q990, Q991, Q992, Q998, Q999, Q87, Q870, Q871, Q872, Q873, Q874, Q875, Q878, Q878 |
| Chronic kidney disease (Delphi: Chronic kidney disease, end stage renal dysfunction) | The best (highest value) of the last 2 eGFR readings is < 60 mL/min | Use of clinical coding and laboratory results | Any Read v2 code ever coded OR application of modified CALIBER “Chronic Kidney Disease” algorithm:  IF egfr_ckdepi coded on or before specified date, THEN IF egfr_ckdepi <60 ml/min on the most recent date (index date) before the specified date AND IF egfr_ckdepi <60 ml/min on any date greater than 90 days BEFORE the index date above THEN classify as having CKD3 or above ELSE the patient is not defined as having CKD stage 3 or above | Any ICD-10 code ever recorded | Any coding as per PC implementation rules OR ICD-10 code ever recorded | \| c811 - end stage renal disease  c2035 - chronic kidney disease \| \| --- \| | \| c813 - end stage renal disease  c2846 - chronic kidney disease \| \| --- \| |
| Chronic liver disease | Read v2 code ever coded | Unchanged | Read 2 code ever recorded | ICD-10 code ever recorded | Read v2 or ICD-10 code ever recorded | c998 - autoimmune liver disease, c1373 - portal HTN, c1082 - liver fibrosis, sclerosis, and cirrhosis, c1265 - alcoholic liver disease, c1645 - oesophageal varices, c1073 – chronic viral hepatits | c1000 - autoimmune liver disease, c1375 - portal HTN, c1084 - liver fibrosis, sclerosis, and cirrhosis, c1267 - alcoholic liver disease, c1647 - oesophageal varices, c1075 chronic viral hepatitis |
| Chronic obstructive pulmonary disease | Read v2 code ever coded | Unchanged | Read 2 code ever recorded | ICD-10 code ever recorded | Read v2 or ICD-10 code ever recorded | c2748 – chronic obstructive pulmonary disease | c2746 - chronic obstructive pulmonary disease |
| Connective tissue disorders | Read v2 code ever coded | Unchanged | Read 2 code ever recorded | ICD-10 code ever recorded | Read v2 or ICD-10 code ever recorded | c815 – giant cell arteritis, c887 – polymyalgia rheumatica, c905 – rheumatoid arthritis,  c912 – systemic lupus erythematosus, c961 - ankylosing spondylosis,  c1249 - juvenile arthritis, c1565 – Sjogren syndrome, c890 - psoriatic arthritis | c817 – giant cell arteritis, c889 – polymyalgia rheumatica, c907 – rheumatoid arthritis,  c914 – systemic lupus erythematosus, c963 - ankylosing spondylosis, c1251 juvenile arthritis, c1567 Sjogren syndrome, c892 psoriatic arthritis |
| Dementia | Read v2 code ever coded | Unchanged | Read 2 code ever recorded | ICD-10 code ever recorded | Read v2 or ICD-10 code ever recorded | c2777 - dementia | c2773 - dementia |
| Depression | Read code coded in last 12 months | Unchanged | Read v2 code in the previous 12-months OR 4 or more antidepressant prescriptions in the previous 12-months | Read v2 code in the previous 12-months | Read v2 or ICD-10 code in the previous 12-months OR 4 or more antidepressant prescriptions in the previous 12-months | c1111 - depression | c1113 - depression |
| Diabetes | Read v2 code ever coded | Unchanged | Read 2 code ever recorded | ICD-10 code ever recorded | Read v2 or ICD-10 code ever recorded | c1120 – diabetes, c1128 - diabetes neurological complications, c1117 - diabetes eye complications | c1122 – diabetes, c1130 - diabetes neurological complications, c1119 - diabetes eye complications |
| Eating disorder | Not included | NA | Read 2 code ever recorded | ICD-10 code ever recorded | Read v2 or ICD-10 code ever recorded | C3252 - eating disorder | c2935 - eating disorder |
| Endometriosis | Not included | NA | Any Read v2 code ever recorded AND age < 55 years | Any ICD-10 code ever recorded AND age < 55 years | Any Read v2 or ICD-10 code ever recorded AND age < 55 years | c1145 - endometriosis | c1147 - endometriosis |
| Epilepsy | Read code ever coded | Unchanged | Read v2 code ever coded AND any antiepileptic (excluding gabapentinoids) prescription in the previous12 months | ICD-10 code ever coded | Read v2 or ICD-10 code ever coded AND any antiepileptic (excluding gabapentinoids) prescription in the previous12 months | c1154 - epilepsy | c1156 - epilepsy |
| Gout | Not included | NA | Read 2 code ever recorded | ICD-10 code ever recorded | Read v2 or ICD-10 code ever recorded | c1191 - gout | c1193 - gout |
| Hearing impairment | Read v2 code ever coded | Unchanged | Read 2 code ever recorded | ICD-10 code ever recorded | Read v2 or ICD-10 code ever recorded | c1102 - hearing loss | c1104 - hearing loss |
| Heart failure | Read v2 code ever coded | Unchanged | Read 2 code ever recorded | ICD-10 code ever recorded | Read v2 or ICD-10 code ever recorded | c1206 - heart failure | c1208 - heart failure |
| Heart valve disorders | Not included | Na | Read 2 code ever recorded | ICD-10 code ever recorded | Read v2 or ICD-10 code ever recorded | c908 - rheumatic heart valve, c1289 - multiple heart valve disorder, c1308 - nonrheumatic aortic valve disorder, c1311 - nonrheumatic mitral valve disorder | c910 - rheumatic heart valve disorder, c1291 - multiple heart valve disorder, c1310 - nonrheumatic aortic valve disorder, c1313 - nonrheumatic mitral valve disorder |
| Hypertension | Read v2 code ever coded | Unchanged | Read 2 code ever recorded | ICD-10 code ever recorded | Read v2 or ICD-10 code ever recorded | c1227 - hypertension | c1229 - hypertension |
| Inflammatory bowel disease | Read v2 code ever coded | Unchanged | Read 2 code ever recorded | ICD-10 code ever recorded | Read v2 or ICD-10 code ever recorded | c1096 - Crohn's disease,  c1621 - ulcerative colitis | c1098 - Crohn's disease,  c1623 - ulcerative colitis |
| Meniere’s disease | Not included | NA | Read 2 code ever recorded | ICD-10 code ever recorded | Read v2 or ICD-10 code ever recorded | c1279 - Meniere's disease | c1281 - Meniere's disease |
| Multiple sclerosis | Read c2 code ever recorded | Unchanged | Read 2 code ever recorded | ICD-10 code ever recorded | Read v2 or ICD-10 code ever recorded | c855 – Multiple Sclerosis | C857 – Multiple sclerosis |
| Osteoarthritis | Read v2 code ever coded | Unchanged | Read 2 code ever recorded | ICD-10 code ever recorded | Read v2 or ICD-10 code ever recorded | c861 - osteoarthritis | c863 - osteoarthritis |
| Osteoporosis | Read v2 code ever coded | Unchanged | Read 2 code ever recorded | ICD-10 code ever recorded | Read v2 or ICD-10 code ever recorded | c1326 - osteoporosis | c1328 - osteoporosis |
| Peripheral arterial disease | Read v2 code ever coded | Unchanged | Read 2 code ever recorded | ICD-10 code ever recorded | Read v2 or ICD-10 code ever recorded | c1349 – peripheral arterial disease | c1351 - peripheral arterial disease |
| Pancreatitis (chronic) | Not included | NA | Read 2 code ever recorded | ICD-10 code ever recorded | Read v2 or ICD-10 code ever recorded | 14CG., J671. | K861, K860 |
| Paralysis | Not included | NA | Read 2 code ever recorded | ICD-10 code ever recorded | Read v2 or ICD-10 code ever recorded | F2411, F141., F241., F2410, F2300, F240.. F2401, F2400, F232., F232.. F240., F242.. F230., F230z, F2301 | G82, G830, G808, G801, G803 |
| Parkinson’s disease | Read v2 code ever coded | Unchanged | Read 2 code ever recorded | ICD-10 code ever recorded | Read v2 or ICD-10 code ever recorded | c896 – Parkinson’s disease | c898 - Parkinson’s disease |
| Peptic ulcer | Read v2 code ever coded | Unchanged | Read 2 code ever recorded | ICD-10 code ever recorded | Read v2 or ICD-10 code ever recorded | c1624 - peptic ulcer | c1626 - peptic ulcer |
| Peripheral neuropathy | Not included | NA | Read 2 code ever recorded | ICD-10 code ever recorded | Read v2 or ICD-10 code ever recorded | c1346 - peripheral neuropathy | c1348 - peripheral neuropathy |
| Post-traumatic stress disorder | Not included | NA | Read 2 code ever recorded | ICD-10 code ever recorded | Read v2 or ICD-10 code ever recorded | E2831, E29y1, Eu431, Eu433, Eu434, ZS7C7 | F431 |
| Schizophrenia | Read code ever coded | Unchanged | Read 2 code ever recorded | ICD-10 code ever recorded | Read v2 or ICD-10 code ever recorded | c1503 - schizophrenia | c1503 - schizophrenia |
| Stroke and transient ischaemic attach (Delphi: stroke, transient ischaemic attack) | Read v2 code ever coded | Unchanged | Read 2 code ever recorded | ICD-10 code ever recorded | Read v2 or ICD-10 code ever recorded | c834 - intracerebral haemorrhage, c837 - ischaemic stroke, c918 - NOS stroke, c921 - subarachnoid haemorrhage, c921, c927 – transient ischaemic attack | c836 - intracerebral haemorrhage, c839 - ischaemic stroke, c920 - NOS stroke, c923 - subarachnoid haemorrhage, c929 transient ischaemic attack |
| Tuberculosis | Not included | NA | Read v2 code in the previous 5 years | ICD-10 code in the previous 5 years | Read v2 of ICD-10 code in the previous 5 years | c924 - tuberculosis | c926 - tuberculosis |
| Thyroid disorders | Read v2 code ever coded | Unchanged | Read 2 code ever recorded | ICD-10 code ever recorded | Read v2 or ICD-10 code ever recorded | C1609 - thyroid | C1611 - thyroid |
| Visual impairment | Read v2 code ever coded | Unchanged | Read 2 code ever recorded | ICD-10 code ever recorded | Read v2 or ICD-10 code ever recorded | c1041 - visual impairment and blindness | c1043 - visual impairment and blindness |
| Venous thromboembolic disease | Not included | NA | Read v2 code in previous 12-months or >1 code ever | ICD-10 code in previous 12-months or >1 code ever | Read v2 or ICD-10 code in previous 12-months or >1 code ever | c880 – pulmonary embolism,  c1657 - deep vein thrombosis | c882 – pulmonary embolism,  c1659 – deep vein thrombosis |

NA = not applicable, condition not included in Barnett et al^1^ analysis

*Read v2 codes truncated to 5-digits for compatibility with SAIL Databank.

**Code lists from the HDR UK Phenotype Library are formatted as cXXX (e.g., c882 is pulmonary embolism) where they are called ‘concepts’ and can be downloaded by searching for the code list/concept number at <https://phenotypes.healthdatagateway.org/concepts/>?

†Available at OpenSAFELY <https://github.com/opensafely/hdruk-os-covid-paeds/commit/6295c353125577798fafe9afa25d882a1b911200>
