# Supplementary material for: Impact of data source choice on multimorbidity measurement: a comparison study of 2.3 million individuals in the Welsh National Health Service
Source: BMC Med. 2023 Aug 15;21:309. doi: 10.1186/s12916-023-02970-z (PMC10426056; doi:10.1186/s12916-023-02970-z)
Supplement: Supplementary file 3 — Additional file 3: Associations between long-term conditions and mortality by data source. [file 12916_2023_2970_MOESM3_ESM.docx]

# Additional File 3. Associations between long-term conditions and mortality by data source.

**Table 1. Logistic regression model where death during 1-year of study follow-up is the outcome, stratified by number of long-term conditions identified in only primary care data.**

|  | Covariate | Crude odds ratio (95% confidence interval) | Adjusted odds ratio (95% confidence interval)* | P (Wald’s test) |
| --- | --- | --- | --- | --- |
| Number of long-term conditions (covariate of interest) | 0 | Ref |  |  |
|  | 1 | 2.72 (2.62,2.82) | 1.32 (1.27,1.37) | < 0.001 |
|  | 2 | 7.09 (6.85,7.33) | 1.95 (1.88,2.02) | < 0.001 |
|  | 3 | 13.97 (13.52,14.44) | 2.61 (2.52,2.71) | < 0.001 |
|  | 4+ | 43.83 (42.63,45.07) | 5.10 (4.94,5.26) | < 0.001 |
| Age group (years) | 0-4 | Ref | Ref | Ref |
|  | 5 - 9 | 0.47 (0.25,0.88) | 0.46 (0.24,0.86) | 0.015 |
|  | 10 - 14 | 1.32 (0.80,2.19) | 1.27 (0.77,2.11) | 0.350 |
|  | 15 - 19 | 2.88 (1.81,4.58) | 2.69 (1.69,4.28) | < 0.001 |
|  | 20 - 29 | 4.94 (3.21,7.60) | 4.23 (2.75,6.51) | < 0.001 |
|  | 30 - 39 | 11.16 (7.31,17.04) | 8.84 (5.79,13.51) | < 0.001 |
|  | 40 - 49 | 25.94 (17.04,39.48) | 18.36 (12.06,27.97) | < 0.001 |
|  | 50 - 59 | 57.07 (37.54,86.76) | 34.14 (22.45,51.93) | < 0.001 |
|  | 60 - 69 | 138.44 (91.11,210.36) | 65.26 (42.91,99.24) | < 0.001 |
|  | 70 - 79 | 362.37 (238.53,550.5) | 136.19 (89.57,207.09) | < 0.001 |
|  | 80 -89 | 1151.68 (758.11,1749.58) | 367.18 (241.46,558.34) | < 0.001 |
|  | 90+ | 3692.64 (2429.47,5612.58) | 1216.99 (799.89,1851.59) | < 0.001 |
| Sex | Men and boys | Ref | Ref | Ref |
|  | Women and girls | 0.96 (0.94,0.97) | 0.69 (0.68,0.71) | < 0.001 |
| Welsh index of multiple deprivation (WIMD) | 1 (least) | Ref | Ref | Ref |
|  | 2 | 1.05 (1.02,1.08) | 0.93 (0.90,0.96) | < 0.001 |
|  | 3 | 1.11 (1.08,1.15) | 0.87 (0.84,0.9) | < 0.001 |
|  | 4 | 1.02 (0.99,1.05) | 0.81 (0.78,0.83) | < 0.001 |
|  | 5 | 1.04 (1.01,1.07) | 0.79 (0.76,0.81) | < 0.001 |
|  | 6 | 1.03 (1.00,1.06) | 0.71 (0.69,0.74) | < 0.001 |
|  | 7 | 1.03 (0.99,1.06) | 0.71 (0.68,0.73) | < 0.001 |
|  | 8 | 1.02 (0.99,1.06) | 0.67 (0.65,0.69) | < 0.001 |
|  | 9 | 0.94 (0.91,0.97) | 0.63 (0.61,0.66) | < 0.001 |
|  | 10 (most) | 0.93 (0.90,0.96) | 0.59 (0.57,0.61) | < 0.001 |

*Ref* – reference category, * adjusted for age, sex, and deprivation.

**Table 2. Logistic regression model where death during 1-year of study follow-up is the outcome, stratified by number of long-term conditions identified in only hospital inpatient data.**

|  | Covariate | Crude odds ratio (95% confidence interval) | Adjusted odds ratio (95% confidence interval)* | P (Wald’s test) |
| --- | --- | --- | --- | --- |
| Number of long-term conditions (covariate of interest) | 0 | Ref | Ref | Ref |
|  | 1 | 3.36 (3.27,3.45) | 1.70 (1.65,1.74) | < 0.001 |
|  | 2 | 8.43 (8.21,8.66) | 2.41 (2.34,2.48) | < 0.001 |
|  | 3 | 15.96 (15.54,16.4) | 3.39 (3.29,3.49) | < 0.001 |
|  | 4+ | 43.10 (42.2,44.03) | 6.95 (6.79,7.12) | < 0.001 |
| Age group (years) | 0-4 | Ref | Ref | Ref |
|  | 5 - 9 | 0.47 (0.25,0.88) | 0.46 (0.24,0.86) | 0.016 |
|  | 10 - 14 | 1.32 (0.80,2.19) | 1.28 (0.77,2.12) | 0.342 |
|  | 15 - 19 | 2.88 (1.81,4.58) | 2.69 (1.69,4.28) | < 0.001 |
|  | 20 - 29 | 4.94 (3.21,7.60) | 4.3 (2.79,6.61) | < 0.001 |
|  | 30 - 39 | 11.16 (7.31,17.04) | 9.33 (6.11,14.25) | < 0.001 |
|  | 40 - 49 | 25.94 (17.04,39.48) | 20.35 (13.36,30.98) | < 0.001 |
|  | 50 - 59 | 57.07 (37.54,86.76) | 38.87 (25.57,59.11) | < 0.001 |
|  | 60 - 69 | 138.44 (91.11,210.36) | 74.09 (48.74,112.61) | < 0.001 |
|  | 70 - 79 | 362.37 (238.53,550.5) | 149.73 (98.52,227.56) | < 0.001 |
|  | 80 -89 | 1151.68 (758.11,1749.58) | 386.49 (254.29,587.41) | < 0.001 |
|  | 90+ | 3692.64 (2429.47,5612.58) | 1299.83 (854.74,1976.71) | < 0.001 |
| Sex | Men and boys | Ref | Ref | Ref |
|  | Women and girls | 0.96 (0.94,0.97) | 0.74 (0.73,0.75) | < 0.001 |
| Welsh index of multiple deprivation (WIMD) | 1 (least) | Ref | Ref | Ref |
|  | 2 | 1.05 (1.02,1.08) | 0.92 (0.89,0.96) | < 0.001 |
|  | 3 | 1.11 (1.08,1.15) | 0.89 (0.86,0.92) | < 0.001 |
|  | 4 | 1.02 (0.99,1.05) | 0.81 (0.78,0.84) | < 0.001 |
|  | 5 | 1.04 (1.01,1.07) | 0.79 (0.76,0.82) | < 0.001 |
|  | 6 | 1.03 (1.00,1.06) | 0.73 (0.71,0.76) | < 0.001 |
|  | 7 | 1.03 (0.99,1.06) | 0.72 (0.69,0.75) | < 0.001 |
|  | 8 | 1.02 (0.99,1.06) | 0.69 (0.67,0.72) | < 0.001 |
|  | 9 | 0.94 (0.91,0.97) | 0.65 (0.62,0.67) | < 0.001 |
|  | 10 | 0.93 (0.90,0.96) | 0.61 (0.59,0.63) | < 0.001 |

*Ref* – reference category, * adjusted for age, sex, and deprivation.

**Table 3. Logistic regression model where death during 1-year of study follow-up is the outcome, stratified by number of long-term conditions identified in linked primary care and hospital inpatient data.**

|  | Covariate | Crude odds ratio (95% confidence interval) | Adjusted odds ratio (95% confidence interval)* | P (Wald’s test) |
| --- | --- | --- | --- | --- |
| Number of long-term conditions (covariate of interest) | 0 | Ref | Ref | Ref |
|  | 1 | 3.59 (3.43,3.76) | 1.73 (1.65,1.81) | < 0.001 |
|  | 2 | 9.25 (8.87,9.65) | 2.68 (2.56,2.80) | < 0.001 |
|  | 3 | 17.82 (17.10,18.56) | 3.58 (3.43,3.74) | < 0.001 |
|  | 4+ | 69.35 (66.92,71.87) | 8.34 (8.02,8.68) | < 0.001 |
| Age group (years) | 0-4 | Ref | Ref | Ref |
|  | 5 - 9 | 0.47 (0.25,0.88) | 0.44 (0.23,0.83) | 0.011 |
|  | 10 - 14 | 1.32 (0.80,2.19) | 1.20 (0.73,1.99) | 0.473 |
|  | 15 - 19 | 2.88 (1.81,4.58) | 2.48 (1.56,3.94) | < 0.001 |
|  | 20 - 29 | 4.94 (3.21,7.60) | 3.64 (2.36,5.60) | < 0.001 |
|  | 30 - 39 | 11.16 (7.31,17.04) | 7.19 (4.71,10.98) | < 0.001 |
|  | 40 - 49 | 25.94 (17.04,39.48) | 13.87 (9.1,21.13) | < 0.001 |
|  | 50 - 59 | 57.07 (37.54,86.76) | 23.79 (15.63,36.21) | < 0.001 |
|  | 60 - 69 | 138.44 (91.11,210.36) | 42.06 (27.64,64) | < 0.001 |
|  | 70 - 79 | 362.37 (238.53,550.50) | 83.22 (54.7,126.61) | < 0.001 |
|  | 80 -89 | 1151.68 (758.11,1749.58) | 217.68 (143.06,331.20) | < 0.001 |
|  | 90+ | 3692.64 (2429.47,5612.58) | 709.59 (466.11,1080.26) | < 0.001 |
| Sex | Women and girls | Ref | Ref | Ref |
|  | Men and boys | 0.96 (0.94,0.97) | 0.7 (0.69,0.71) | < 0.001 |
| Welsh index of multiple deprivation (WIMD) | 1 (least) | Ref | Ref | Ref |
|  | 2 | 1.05 (1.02,1.08) | 0.93 (0.90,0.97) | < 0.001 |
|  | 3 | 1.11 (1.08,1.15) | 0.88 (0.85,0.92) | < 0.001 |
|  | 4 | 1.02 (0.99,1.05) | 0.81 (0.79,0.84) | < 0.001 |
|  | 5 | 1.04 (1.01,1.07) | 0.80 (0.77,0.83) | < 0.001 |
|  | 6 | 1.03 (1.00,1.06) | 0.73 (0.71,0.76) | < 0.001 |
|  | 7 | 1.03 (0.99,1.06) | 0.72 (0.69,0.75) | < 0.001 |
|  | 8 | 1.02 (0.99,1.06) | 0.69 (0.67,0.72) | < 0.001 |
|  | 9 | 0.94 (0.91,0.97) | 0.65 (0.62,0.67) | < 0.001 |
|  | 10 | 0.93 (0.90,0.96) | 0.61 (0.59,0.63) | < 0.001 |

*Ref* – reference category, * adjusted for age, sex, and deprivation.
