# Supplementary material for: Impact of data source choice on multimorbidity measurement: a comparison study of 2.3 million individuals in the Welsh National Health Service
Source: BMC Med. 2023 Aug 15;21:309. doi: 10.1186/s12916-023-02970-z (PMC10426056; doi:10.1186/s12916-023-02970-z)
Supplement: Supplementary file 4 — Additional file 4: Measures of concordance of long-term conditions using primary care and hospital inpatient data. [file 12916_2023_2970_MOESM4_ESM.docx]

# Additional File 4. Measures of concordance of long-term conditions using primary care and hospital inpatient data.

Percent ascertainment using only primary care (PC) and only hospital inpatient (HI) data when compared with linked primary care and hospital inpatient (PC-HI) data. Dark grey bars represent the only HI/linked PC-HI ratio, light grey bars represent the only PC/PC-HI linked ratio. Percent agreement between data sources (where the same individuals were identified in both PC and HI data) is marked by black diamonds. Conditions are grouped by ICD-10 chapter.
