# Supplementary material for: Impact of data source choice on multimorbidity measurement: a comparison study of 2.3 million individuals in the Welsh National Health Service
Source: BMC Med. 2023 Aug 15;21:309. doi: 10.1186/s12916-023-02970-z (PMC10426056; doi:10.1186/s12916-023-02970-z)
Supplement: Supplementary file 5 — Additional file 5: Measures of concordance of long-term conditions using different data sources. [file 12916_2023_2970_MOESM5_ESM.docx]

# Additional File 5. Measures of concordance of long-term conditions using different data sources.

| **ICD-10 chapter** | **Long-term condition** | **PC/HI**  **Ratio (95% CI)** | **PC/linked PC-HI**  **Ratio (95%CI)** | **HI/linked PC-HI**  **Ratio (95%CI)** | **Found only in PC** | **Found only in HI** | **Agreement (same individuals identified in PC and HI data)**  **Number (%)** | **Total** | **Kappa statistic*** |
| --- | --- | --- | --- | --- | --- | --- | --- | --- | --- |
| I - Certain infectious and parasitic diseases | Tuberculosis | 3.2 (2.7,3.6) | 85.0 (82.7, 87.1) | 26.9 (24.2,29.7) | 753 (73.1) | 154 (15.0) | 123 (11.9) | 1030 | 0.21 |
| II - Neoplasms | Cancer | 1.0 (1.0,1.0) | 78.6 (78.2,78.0) | 75.6 (75.2,75.9) | 13031 (24.4) | 11410 (21.4) | 28874 (54.2) | 53315 | 0.69 |
| III - Diseases of blood/ blood-forming organs | Anaemia | 2.3 (2.3,2.4) | 76.0 (75.6,76.4) | 32.5 (32.0, 32.9) | 26702 (67.5) | 9495 (24) | 3338 (8.4) | 39535 | 0.28 |
| IV - Endocrine, nutritional and metabolic diseases | Cystic fibrosis | 2.1 (1.9, 2.4) | 92.6 (90.8,94.1) | 43.2 (40.3,46.3) | 614 (56.7) | 80 (7.4) | 388 (35.9)^a^ | 1082 | 0.52 |
|  | Thyroid disorders | 1.9 (1.9,1.9) | 94.9 (94.7,95.0) | 50.1 (49.8,50.4) | 63735 (49.9) | 6559 (5.1) | 57482 (45.0) | 127776 | 0.61 |
|  | Addison's disease | 0.8 (0.7,0.8) | 63.6 (61.3,65.9) | 81.2 (79.2,83.0) | 318 (18.8) | 614 (36.4) | 757 (44.8) | 1689 | 0.62 |
|  | Diabetes | 1.4 (1.4,1.5) | 93.7 (93.6,93.9) | 64.7 (64.5,64.9) | 56284 (35.3) | 9968 (6.2) | 93243 (58.5) | 159495 | 0.72 |
| V - Mental and behavioural disorders | PTSD | 4.6 (4.5,4.7) | 90.0 (89.5,90.5) | 19.5 (18.8,20.1) | 10740 (80.5) | 1332 (10) | 1268 (9.5) | 13340 | 0.17 |
|  | Depression | 14.2 (14.0,14.4) | 98.2 (98.1,98.2) | 6.9 (6.8,7.0) | 289978 (93.1) | 5603 (1.8) | 15937 (5.1) | 311518 | 0.08 |
|  | Eating disorder | 6.6 (6.3,6.9) | 95.4 (95.0,95.8) | 14.4 (13.8,15.1) | 10610 (85.6) | 569 (4.6) | 1220 (9.8) | 12399 | 0.18 |
|  | Anxiety | 5.1 (5.0,5.1) | 87.4 (87.1,87.6) | 17.2 (17.0,17.4) | 87770 (82.8) | 13402 (12.6) | 4852 (4.6) | 106024 | 0.30 |
|  | Autism | 2.9 (2.8,3.0) | 91.3 (90.9,91.8) | 31.4 (30.7,32.1) | 10728 (68.6) | 1352 (8.6) | 3555 (22.7) | 15635 | 0.37 |
|  | Alcohol & substance misuse | 2.2 (2.2,2.2) | 84.7 (84.5,84.9) | 38.6 (38.2,38.9) | 59358 (61.4) | 14780 (15.3) | 22472 (23.3) | 96610 | 0.78 |
|  | Bipolar affective disorder | 2.0 (2.0,2.1) | 86.5 (86.0,87.0) | 42.1 (41.4,42.9) | 8985 (57.8) | 2096 (13.5) | 4452 (28.7) | 15533 | 0.44 |
|  | Dementia | 1.8 (1.8,1.9) | 87.0 (86.5,87.5) | 47.8 (47.2,48.5) | 9482 (52.2) | 2354 (13) | 6335 (34.9) | 18171 | 0.51 |
|  | Schizophrenia | 1.5 (1.5,1.7) | 84.1 (83.5,84.7) | 54.8 (54.0,55.5) | 7597 (45.2) | 2668 (15.9) | 6527 (38.9) | 16792 | 0.56 |
| VI - Diseases of the nervous system | Peripheral neuropathy | 3.2 (3.1,3.2) | 84.7 (84.4,85.0) | 26.7 (26.3,27.1) | 41946 (73.3) | 8764 (15.3) | 6516 (11.4) | 57226 | 0.20 |
|  | Parkinson's disease | 1.3 (1.3,1.4) | 84.4 (83.4,85.3) | 62.4 (61.2,63.6) | 2248 (37.1) | 936 (15.4) | 2799 (46.2) | 6062 | 0.64 |
|  | Multiple sclerosis | 1.3 (1.3,1.4) | 91.9 (91.2,92.6) | 68.5 (67.2,69.8) | 1616 (31.5) | 418 (8.1) | 3102 (60.4) | 5136 | 0.75 |
|  | Epilepsy† | 0.7 (0.7,0.7) | 80.1 (79.5,80.6) | N/A | N/A | N/A | N/A | N/A | N/A |
|  | Paralysis | 0.7 (0.7,0.7) | 53.5 (51.8,55.2) | 76.5 (75.0,78.0) | 796 (23.5) | 1571 (46.5) | 1015 (30.0) | 3382 | 0.46 |
| VII - Diseases of the eye and adnexa | Visual impairment | 3.5 (3.4,3.6) | 87.9 (87.4,88.3) | 25.2 (24.6,25.8) | 15702 (74.8) | 2540 (12.1) | 2748 (13.1) | 20990 | 0.23 |
| VIII - Diseases of the ear and mastoid process | Hearing impairment | 9.1 (8.9,9.2) | 95.6 (95.6,95.7) | 10.5 (10.4,10.7) | 193817 (89.4) | 9437 (4.4) | 13428 (6.2) | 216682 | 0.10 |
|  | Meniere's disease | 8.7 (8.4,9.1) | 96.6 (96.3,96.8) | 11.2 (10.7,11.5) | 20861 (88.9) | 800 (3.4) | 1800 (7.7) | 23461 | 0.14 |
| IX - Diseases of the circulatory system | Venous thromboembolism | 3.3 (3.2,3.3) | 93.8 (93.6,94.0) | 28.5 (28.1,28.9) | 39250 (71.5) | 3398 (6.2) | 12259 (22.3) | 54907 | 0.36 |
|  | Peripheral artery disease | 2.5 (2.4,2.5) | 87.4 (87.2,87.8) | 35.2 (34.8,35.8) | 22857 (64.7) | 4440 (12.6) | 8006 (22.7) | 35303 | 0.36 |
|  | Stroke and TIA | 1.6 (1.6,1.8) | 89.1 (88.9,89.4) | 54.1 (53.8,54.5) | 32036 (45.8) | 7597 (10.9) | 30256 (43.3) | 69889 | 0.60 |
|  | Hypertension | 1.4 (1.4, 1.4) | 88.1 (88.0,88.2) | 63.7 (63.6,63.9) | 165692 (36.3) | 54365 (11.9) | 236630 (51.8) | 456687 | 0.64 |
|  | Heart failure | 1.0 (1.0,1.0) | 68.8 (68.3,69.2) | 67.0 (66.6,67.4) | 15009 (33) | 14213 (31.2) | 16286 (35.8) | 45508 | 0.52 |
|  | Coronary artery disease | 0.9 (0.9,0.9) | 79.2 (79.0,79.5) | 83.6 (83.4,83.8) | 20909 (16.4) | 26487 (20.7) | 80254 (62.9) | 127650 | 0.76 |
|  | Heart valve disorders | 0.9 (0.9,0.9) | 63.7 (63.3,64.1) | 67.8 (67.4,68.2) | 14779 (32.2) | 16668 (36.3) | 14484 (31.5) | 45931 | 0.47 |
|  | Arrythmia | 1.0 (0.9,1.0) | 77.6 (77.3,77.8) | 80.4 (80.1,80.7) | 17380 (19.6) | 19905 (22.4) | 51461 (58.0) | 88746 | 0.73 |
|  | Aneurysm | 0.5 (0.5,0.5) | 41.5 (40.3,42.8) | 82.2 (81.2,83.1) | 1095 (17.8) | 3593 (58.5) | 1455 (23.7) | 6143 | 0.38 |
| X - Diseases of the respiratory system | Bronchiectasis | 1.6 (1.6, 1.7) | 82.7 (82.0,83.4) | 50.9 (50.0,51.9) | 5363 (49.1) | 1890 (17.3) | 3677 (33.6) | 10930 | 0.50 |
|  | COPD | 1.2 (1.1, 1.2) | 76.2 (75.9,76.5) | 65.2 (65.0,65.5) | 29260 (34.8) | 19992 (23.8) | 34823 (41.4) | 84075 | 0.58 |
|  | Asthma† | 1.1 (1.1,1.1) | 73.3 (73.1,73.4) | N/A | N/A | N/A | N/A | N/A | N/A |
| XIII - Diseases of the musculoskeletal system | Gout | 5.3 (5.2,5.4) | 97.3 (97.2,97.4) | 18.4 (18.1,18.6) | 69638 (81.6) | 2328 (2.7) | 13364 (15.7) | 85330 | 0.26 |
|  | Connective tissue disorders | 1.8 (1.8,1.8) | 87.3 (87.0,87.5) | 47.8 (47.4,48.2) | 33701 (35.6) | 38226 (40.4) | 22644 (23.9) | 94571 | 0.51 |
|  | Osteoarthritis | 1.7 (1.7,1.7) | 82.9 (82.7,83.0) | 48.7 (48.5,48.8) | 150777 (51.3) | 50264 (17.1) | 92637 (31.5) | 293678 | 0.44 |
|  | Osteoporosis | 0.9 (0.8,0.9) | 55.8 (55.5,56.1) | 64.6 (64.3,64.9) | 34972 (35.4) | 43633 (44.2) | 20072 (20.3) | 98677 | 0.32 |
| XI – Diseases of the digestive system | Peptic ulcer | 1.5 (1.4, 1.5) | 74.6 (74.3,75.0) | 51.0 (50.5,51.4) | 22198 (49) | 11505 (25.4) | 11590 (25.6) | 45293 | 0.40 |
|  | Chronic liver disease | 1.4 (1.4,1.4) | 77.1 (76.5,77.7) | 55.2 (54.4,55.9) | 8022 (44.8) | 4099 (22.9) | 5782 (32.3) | 17903 | 0.49 |
|  | Inflammatory bowel disease | 1.1 (1.1,1.1) | 83.3 (82.8,83.7) | 75.7 (75.1,76.2) | 5850 (24.3) | 4021 (16.7) | 14166 (58.9) | 24037 | 0.74 |
|  | Chronic pancreatitis | 0.8 (0.8,0.9) | 59.8 (58.2,61.5) | 71.2 (69.7,72.7) | 980 (28.8) | 1367 (40.1) | 1058 (31.1) | 3405 | 0.47 |
| XIV - Diseases of the genitourinary system | Chronic kidney disease | 2.0 (2.0,2.0) | 84.1 (83.9,84.3) | 41.6 (41.3,41.9) | 76671 (58.4) | 20855 (15.9) | 33822 (25.7) | 131348 | 0.39 |
|  | Endometriosis | 0.9 (0.9,0.9) | 67.5 (66.8,68.2) | 73.6 (72.9,74.2) | 4775 (26.4%) | 5870 (32.5) | 7435 (41.1) | 18080 | 0.58 |
| XVII - Congenital malformations | Congenital disease | 2.1 (2.0,2.2) | 89.1 (88.4,89.7)  vs | 42.8 (41.8, 43.9) | 4674 (57.2%) | 894 (10.9) | 2608 (32.0) | 8176 | 0.48 |

*Interpretation of concordance using Kappa statistic as follows (marked with blue shading on a scale from white for lowest to darkest blue for highest concordance): ≤0.20 = slight (white), 0.21 – 0.40 = fair, 0.41 – 0.60 = moderate, 0.61 – 0.80 = substantial, 0.81 – 1.00 = almost perfect (dark blue).

†Primary care (PC) ascertainment of epilepsy and asthma is Read v2 code ever coded AND prescription, hospital inpatient (HI) is ascertainment of ICD-10 code ever recorded, and linked PC-HI is any Read v2 or ICD-10 code ever recorded AND a prescription. Therefore, calculation of mutually exclusive groups was not possible given that HI prevalence was unconstrained and linked PC-HI prevalence was constrained by the presence of prescribing data.
